# Satisfaction With Patient Engagement and Self-Reported Depression Among Hospitalized Patients: A Propensity-Score Matching Analysis

**DOI:** 10.3389/fpsyt.2022.751412

**Published:** 2022-03-09

**Authors:** Richard Huan Xu, Ling-ming Zhou, Eliza Lai-yi Wong, Jinghui Chang, Dong Wang

**Affiliations:** ^1^Department of Rehabilitation Sciences, The Hong Kong Polytechnic University, Kowloon, Hong Kong SAR, China; ^2^JC School of Public Health and Primary Care, The Chinese University of Hong Kong, Shatin, Hong Kong SAR, China; ^3^School of Health Management, Southern Medical University, Guangzhou, China; ^4^Institute of Health Management, Southern Medical University, Guangzhou, China

**Keywords:** patient engagement, depression, public healthcare system, China, propensity score match

## Abstract

**Background:**

Depression is common among hospitalized patients and poses a significant threat to their quality of life. Patient engagement (PE) in healthcare has been shown to be associated with positive health outcomes. However, the relationship between PE and depression among hospitalized patients, with and without chronic conditions, has not yet been explored. This study aimed to investigate the association between patients' satisfaction with PE and self-reported depression in Chinese public hospitals.

**Method:**

A multi-centered, cross-sectional survey was conducted in seven tertiary-level public hospitals in Guangdong province, China. Twelve items from a patient-centered care questionnaire and the Patient Health Questionnaire 2-item version were used were used to assess patients' satisfaction with PE and self-reported depression, respectively. Propensity score matching (PSM) approach was used to reduce selection bias and potential baseline differences between patients with and without chronic conditions. The relationship between satisfaction with PE and depression was assessed, using univariate and multivariate logistic regression analyses, respectively.

**Results:**

A total of 1,974 hospitalized patients participated in the survey. After the PSM procedure, 604 patients were assigned to the chronic condition group, and another 604 patients were successfully matched in the comparison group with no differences in sex, age, educational level, and PE-related characteristics. Univariate logistic regression analysis indicated that high satisfaction with PE-related approaches significantly decreased the probability of developing depressive status. Multivariate logistic regression analysis further indicated that, after adjusting all PE-related approaches, “patient education” and “involvement in discharge planning” could significantly decrease the probability of patients developing depression.

**Conclusions:**

Our results indicate that encouraging PE and improving patients' satisfaction with PE interventions in clinical practice led to improved mental health outcomes among hospitalized patients in China.

## Introduction

As proven, patient engagement (PE) in healthcare is associated with positive health outcomes, good patient-provider communication, quality healthcare, and decreased costs (1, 2). It is important to attune patients, families, and medical professionals to each other's needs and aspirations (3, 4). Although encouraging patients to actively engage in their own health care has recently been recognized as a rhetoric in professional guidelines and political documents, empirical evidence regarding PE interventions improving health outcomes in clinical routine has rarely been identified (5). PE poses challenges for both medical providers and patients. Studies have identified that many doctors overestimate their communication ability, and demonstrate a poor understanding of their patients' preferences for involvement in decision making, especially with first-time patients (6). Other studies have observed that patients were not content even when their doctors considered engaging them in adequate or even excellent decision-making (7). Understanding the discrepancy in the concept of PE between patients and providers may lead to distinct expectations of healthcare outcomes. Recently, instruments such as the Patient Health Engagement Scale (8) and Patient Engagement Index Questionnaire (9) have been developed to measure PE, but their performance has not been confirmed in the broader community. Without developing a reliable pathway between encouraging PE and improving health outcomes, meaningful PE cannot be expected in clinical practice.

Depression and its sequelae are common and pose a great threat to the quality of life and well-being among hospitalized patients. AlBekairy et al. reported that the majority of patients with diabetes mellitus developed moderate and severe anxiety or depression during hospitalization (10). Alexandri et al. found that nearly 52 and 38% of hospitalized patients with acute myocardial infarction demonstrated high levels of anxiety and depression, respectively (11). Another cohort study also indicated that comorbid depression in American hospitalized patients with failed back surgery syndrome increased by 3% within 5 years (12). Some empirical evidence further indicates that depression adversely affects patients' overall survival (13), frequency of hospital admission (14), non-adherence to treatment (15), poor treatment response (16), malnutrition (17), and all-cause mortality (18). Depression can also lead to a significantly high rate of suicide among hospitalized patients (19).

A hospital is widely recognized as an unpleasant and isolating place, fraught with uncertainties and high levels of depression (20). Recently, engagement efforts to improve patients' experiences with mental health services are frequently reported (21). PE is identified as an approach to support the autonomy and self-determination of patients with psychological distress, so that they can reclaim their preferences and values as persons and improve their mental well-being (22). However, the benefits of PE in decreasing depression among hospitalized patients in China have not been reported, thus affecting strategy development and policies to improve people-centered integrated care. Additionally, studies have confirmed that PE is one of the best tools for managing chronic diseases (23, 24). Ensuring that patients are involved in and knowledgeable about their health is vital when addressing a chronic illness. Actively engaged patients are believed to be more adhered and self-disciplined (24, 25), which facilitates them to embrace both clinical and psychosocial perspectives to manage chronic conditions and actively alter their lifestyle to diminish the harmful effects on health outcomes (26).

Previous research has found that patients with chronic disease can benefit from actively engaging in clinical decision making, which in turn can improve their knowledge of disease and understanding of risk perceptions (27–29). Actively engaged patients, who are more comfortable with treatment, are less likely to opt for major surgery, show better treatment adherence, with improved confidence and coping skills, and report improved health behaviors (30, 31). Further, there is a significant association between gender, age, and educational level and prevalence of chronic conditions. Moreover, patients with a range of chronic conditions are also more likely to have severe depression (32). As chronic diseases become more prevalent, it is essential to control the associated confounding effects of chronic conditions to evaluate the relationship between PE and depression in Chinese hospitalized patients. In this study, we assumed that there is a significant relationship between patients' improved satisfaction with PE and a decrease in self-reported depression. Thus, the objective was to investigate such relationship based on propensity score matching (PSM) analysis, which can reduce the selection bias and potential baseline differences between patients with and without chronic conditions.

## Methods

### Participants and Data Collection

Data used for this analysis were obtained from a patient-centered care survey conducted in seven tertiary-level public hospitals in five cities in Guangdong province, China, from November 2019 to January 2020. Patients who visited six clinical departments (orthopedics, oncology, cardiovascular disease, surgery, obstetrics and gynecology, and hematology) in each hospital were approached by investigators and invited to complete a questionnaire about their demographics, socioeconomic status, health status, quality of life, and experience in using healthcare services. A research nurse from each department, who collaborated with the research team, was appointed as the person responsible for setting up the survey schedule and confirming the participant's eligibility. The inclusion criteria were as follows: (1) ≥18 years, (2) no cognitive problems, (3) able to complete the questionnaire independently, and (4) able to provide informed consent. Of all patients, 2,782 met our criteria and were, therefore, invited to participate in the survey and complete the questionnaire in their ward during the surveying period. Finally, a total of 2,287 (response rate = 82.2%) patients successfully completed and returned the questionnaire (response rate for each hospital is provided in the Supplementary Table 1). After removing missing values, data on sociodemographic status and PE-related outcomes from 1,974 patients were collected for analysis. The study protocol and informed consent were approved by the Ethics Committee of the Second Affiliated Hospital of Guangzhou Medical University (Ref ID: 2019-ks-28). Written informed consent was obtained from all the participants.

### Measures

#### Information Regarding Demographics and Chronic Conditions

Patients' background information, including gender, age, educational level, and family registry (urban and rural resident), as well as their chronic disease from a condition list, was collected.

#### Patient Satisfaction in PE

In this study, the outcomes of 12 items from three sections (shared decision-making, doctor-patient communication, and patient experience) of the patient centered care questionnaire were elicited to measure patients' satisfaction with PE. They were: (1) medical providers provide me with multiple treatment-related options; (2) medical providers patiently listen to my story; (3) medical providers respect my opinions in decision-making; (4) medical providers carefully discuss the treatment plan with me; (5) medical providers understand my concerns and preferences in making decisions; (6) medical providers comfort me when I feel stressed; (7) communication time with medical providers is sufficient; (8) medical providers clearly inform me of the progress of my disease; (9) medical providers seek my approval before treatment starts; (10) medical providers answer my questions timeously; (11) medical providers offer me sufficient patient education; and (12) medical providers involve me in determining discharge planning. Patients were asked to rate their satisfaction with each item on a scale ranging from 1 to 10, with 1 indicating being very dissatisfied and 10 indicating being very satisfied.

#### Self-Reported Depression

Patient Health Questionnaire 2-item version (PHQ-2), a rapid screening instrument, was used to measure patients' depressive status. PHQ-2 includes the first two items of the PHQ-9 (33). The patients were asked to recall the frequency of depressed mood and anhedonia over the past 2 weeks. A PHQ-2 score ≥3 (0–6) indicates a depressed mood. The psychometric properties of the Chinese version of the PHQ-2 have been reported by Liu et al. (34). In this study, the internal consistency reliability of the PHQ-2 was acceptable (Cronbach's Alpha = 0.73).

#### Covariates and PSM Approach

All participants were divided into two groups: those with chronic conditions and those without. To control for the effect of potential confounders on selection bias, a propensity score matched pair method with 1-to-1 nearest neighbor matching, without replacement, and a caliper of 0.01, was used (35). The multiple logistic regression model with covariates was used to estimate propensity scores to gauge the probability of participants from different groups. In this study, the unbalanced conditions between the two groups (with/without chronic conditions) were controlled by using PSM with covariate adjustment, that generated an equal number of matched pairs of participants with and without chronic conditions, having no differences in sex, age, educational level, and the outcomes of PE-related items.

### Statistical Analysis

Descriptive analysis was used to describe the mean and standard deviation (SD) of PE item scores, patients' age, and the proportion of their sex and educational levels. The Wilcoxon rank sum test for continuous variables (score of PE items, age, and physical health status) and chi-squared test (sex and educational level) for categorical variables, were used to assess the differences in participants' responses between groups with and without chronic conditions, before and after PSM, respectively. Univariate and multivariate logistic regression models were developed, and the odds ratios (ORs) and 95% confidence intervals (95% CI) were calculated to evaluate the probability of self-reported depression associated with patients' satisfaction with PE after PSM. In addition, associations between self-reported depression and satisfaction with PE among patients with the three most reported diseases in this sample (cancer, cardiovascular disease, and orthopedic problems) and stratified by family registry, were estimated. All statistical analyses were performed by R software (36), and the PSM was implemented using the package “*MatchIt*.” Statistical significance was set at *p* < 0.05.

## Results

**Figure 1:**
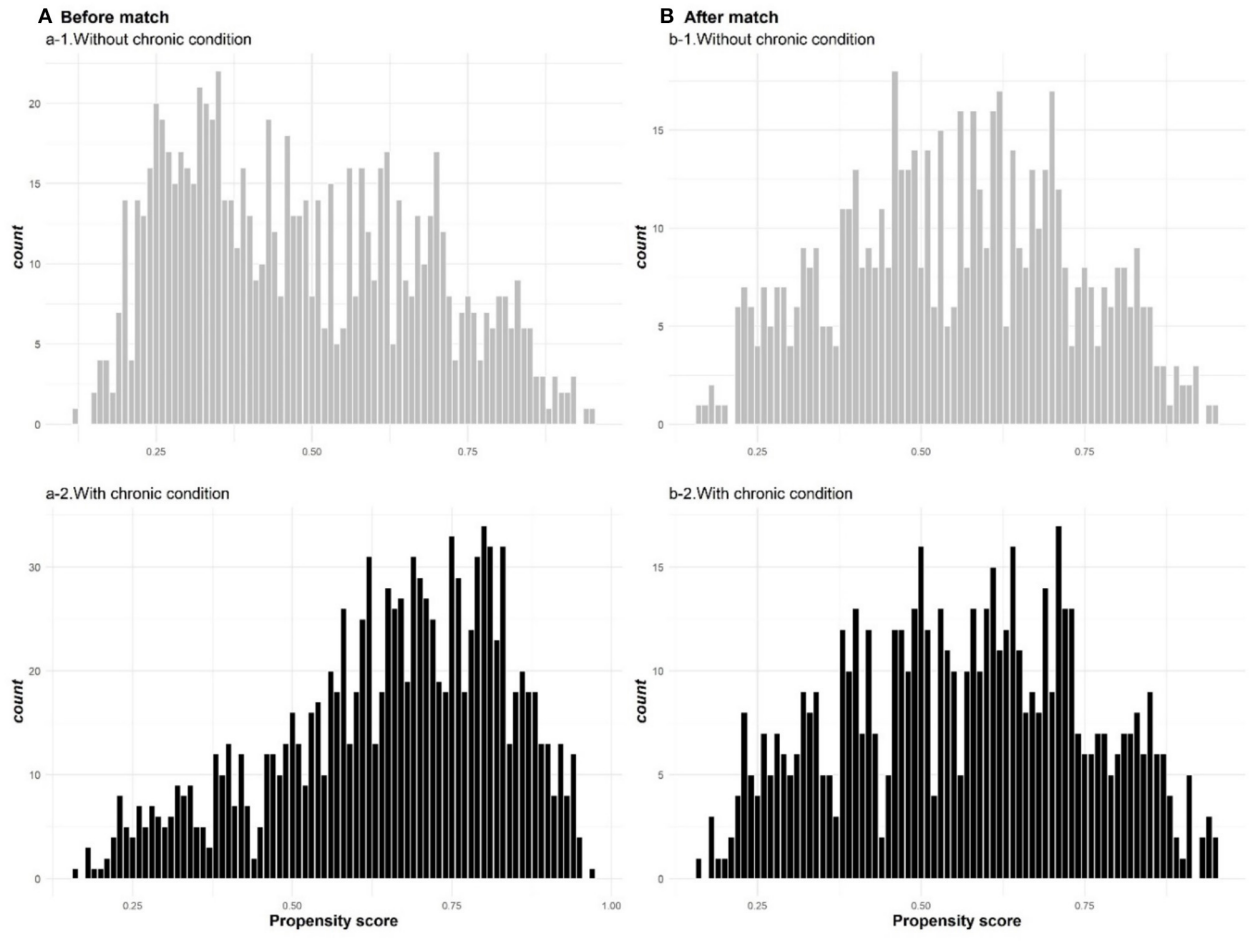
The distribution of propensity scores for participants with and without chronic conditions before **(A)** and after **(B)** PSM.

Figure 1 illustrates the distribution of propensity scores for participants with and without chronic conditions both before and after PSM. Figure 1A presents the histograms of unbalanced propensity score distribution at the baseline (before PSM), indicating that a high proportion of patients without chronic conditions reported a propensity score of −0.3. For patients with chronic conditions, however, the majority reported a propensity score of −0.75. Figure 1B illustrates that after PSM, the propensity score distribution for patients with and without chronic conditions was balanced.

Table 1 indicates that a total of 1,974 patients met the eligibility criteria, of which 1,146 reported having chronic conditions and 828 reported not. After the PSM procedure, 604 patients were assigned to the chronic condition group and successfully matched with another 604 patients in the comparison group (without chronic conditions). In general, approximately 53 and 20.5% of after-matched patients with chronic conditions were male and had completed primary education, respectively. After-matched patients from the group with chronic conditions demonstrated higher satisfaction with PE than patients from the group without chronic conditions, while all comparisons were statistically insignificant. However, after-matched patients without chronic conditions reported a better physical health status than patients with chronic conditions (Mean_non−chronic_ = 80.43, Mean_chronic_ = 73.23, *p* < 0.001).

**Table 1:** Patients' characteristics, satisfaction in medical care engagement and physical health status before and after propensity score matching.

	**Before Matching (*****n** **=*** **1,974)**			**After Matching (*****n** **=*** **1,208)**		
	**Chronic condition**	**No chronic condition**	***p*** **-value**	**Chronic condition**	**No chronic condition**	***p*** **-value**
	**(*n =* 1,146)**	**(*n =* 828)**		**(*n =* 604)**	**(*n =* 604)**	
**Sex**						
Male	573 (50)	386 (46.6)	0.14	320 (53)	316 (52.3)	0.82
Female	573 (50)	442 (53.4)		284 (47)	288 (47.7)	
Age	55.05 (15.12)	42.01 (14.72)	<0.001	48.09 (14.62)	46.94 (14.01)	0.21
**Educational level**						
Primary	285 (24.9)	120 (14.5)	<0.001	124 (20.5)	114 (18.9)	0.77
Secondary	744 (64.9)	515 (62.2)		392 (64.9)	399 (66.1)	
Tertiary or above	117 (10.2)	193 (23.3)		88 (14.6)	91 (15.1)	
Informing all possible options	8.69 (1.75)	8.57 (1.74)	0.02	8.66 (1.66)	8.45 (1.9)	0.14
Listening to my story	8.85 (1.63)	8.7 (1.64)	0.02	8.78 (1.56)	8.58 (1.81)	0.13
Respecting my preferences	8.92 (1.57)	8.79 (1.55)	0.01	8.84 (1.5)	8.7 (1.73)	0.27
Discussing medical plans with me	8.87 (1.63)	8.77 (1.66)	0.14	8.8 (1.62)	8.63 (1.8)	0.09
Understanding my thoughts and concerns	8.8 (1.72)	8.74 (1.66)	0.16	8.78 (1.64)	8.58 (1.86)	0.12
Comforting me when I feel stressed	8.89 (1.6)	8.72 (1.62)	0.005	8.79 (1.6)	8.62 (1.81)	0.17
Communication time is satisfactory	8.9 (1.58)	8.75 (1.6)	0.007	8.82 (1.57)	8.67 (1.75)	0.28
Telling me the disease progress in a clear way	8.96 (1.56)	8.85 (1.53)	0.02	8.9 (1.51)	8.76 (1.73)	0.45
Seeking my approval before treatment start	9.11 (1.44)	8.96 (1.43)	0.004	9.02 (1.36)	8.88 (1.66)	0.41
Answering my questions timely	9 (1.55)	8.85 (1.55)	0.01	8.94 (1.48)	8.79 (1.72)	0.25
Providing sufficient patient education	8.36 (1.92)	8.33 (1.82)	0.25	8.38 (1.8)	8.15 (2.03)	0.07
Involving me in discharge planning	8.77 (1.68)	8.71 (1.62)	0.2	8.75 (1.61)	8.56 (1.81)	0.05
Physical health status (0–100)	72.56 (17.47)	80.68 (14.08)	<0.001	73.23 (16.03)	80.43 (13.89)	<0.001

Table 2 presents the results of the univariate and multivariate logistic regression analyses that predict patients' depressive status by their satisfaction with PE. For the univariate analyses, patients who were highly satisfied with PE were more likely to report a non-depressive status, especially for items ‘Comforting me when I feel stressed' (OR = 1.177, 95% CI = 1.097–1.264), “Answering my questions timeously” (OR = 1.17, 95% CI = 1.086–1.262), and “Providing sufficient patient education” (OR = 1.177, 95% CI = 1.105–1.25). Multivariate regression analysis indicated that patients who were not satisfied with the patient education program were more likely to report a depressive status (OR = 1.205, 95% CI = 1.079–1.348). Likewise, the stratified analysis indicated that urban and rural patients who were satisfied with patient education (OR = 1.307, 95%CI = 1.102–1.558) and involvement in discharge planning (OR = 1.27, 95%CI = 1.05–1.54) tended to report a low level of depression, respectively. The results of the logistic regression-assessed relationship between PE and depression adjusted by patients' chronic conditions without PSM (*n* = 1,974) are provided in the Supplementary Table 2.

**Table 2:** Univariate and multivariate logistic regression analyses for predictors of depressive status in 1,208 subjects after propensity score matching.

	**Univariate analysis**	**Multivariate analysis**		
	**Overall**	**Overall**	**Urban (*n =* 573)**	**Rural (*n =* 635)**
	**OR (95% C.I.)**	**OR (95% C.I.)**	**OR (95% C.I.)**	**OR (95% C.I.)**
Informing all possible options	1.145 (1.069–1.225) ^***^	1.041 (0.904–1.197)	0.901 (0.682–1.177)	1.115 (0.938–1.328)
Listening to my story	1.138 (1.059–1.223) ^***^	0.911 (0.753–1.1)	0.853 (0.63–1.151)	0.944 (0.724–1.223)
Respecting my preferences	1.168 (1.084–1.259) ^***^	1.095 (0.901–1.334)	1.004 (0.732–1.384)	1.157 (0.891–1.51)
Discussing medical plans with me	1.146 (1.068–1.229) ^***^	1.002 (0.835–1.198)	1.16 (0.855–1.568)	0.944 (0.742–1.191)
Understanding my thoughts and concerns	1.154 (1.077–1.236) ^***^	1.048 (0.888–1.232)	1.067 (0.814–1.394)	1 (0.804–1.235)
Comforting me when I feel stressed	1.177 (1.097–1.264) ^***^	1.139 (0.983–1.318)	1.079 (0.85–1.362)	1.175 (0.961–1.438)
Communication time is satisfactory	1.147 (1.066–1.234) ^***^	0.955 (0.787–1.156)	0.913 (0.656–1.26)	1.004 (0.775–1.294)
Telling me the disease progress in a clear way	1.148 (1.066–1.237) ^***^	0.959 (0.778–1.179)	1.004 (0.714–1.401)	0.945 (0.712–1.254)
Seeking my approval before treatment start	1.143 (1.055–1.237) ^***^	0.919 (0.75–1.123)	0.901 (0.647–1.253)	0.925 (0.707–1.206)
Answering my questions timely	1.17 (1.086–1.262) ^***^	1.107 (0.931–1.317)	1.138 (0.851–1.516)	1.105 (0.881–1.389)
Providing sufficient patient education	1.177 (1.105–1.255) ^***^	1.205 (1.079–1.348) ^***^	1.307 (1.102–1.558) ^**^	1.137 (0.978–1.321)
Involving me in discharge planning	1.107 (1.031–1.188) ^***^	1.182 (1.03–1.362) ^*^	0.939 (0.751–1.162)	1.27 (1.05–1.54) ^*^

* *p < 0.05;*

** *p < 0.01;*

*** *p < 0.001*.

**Figure 2:**
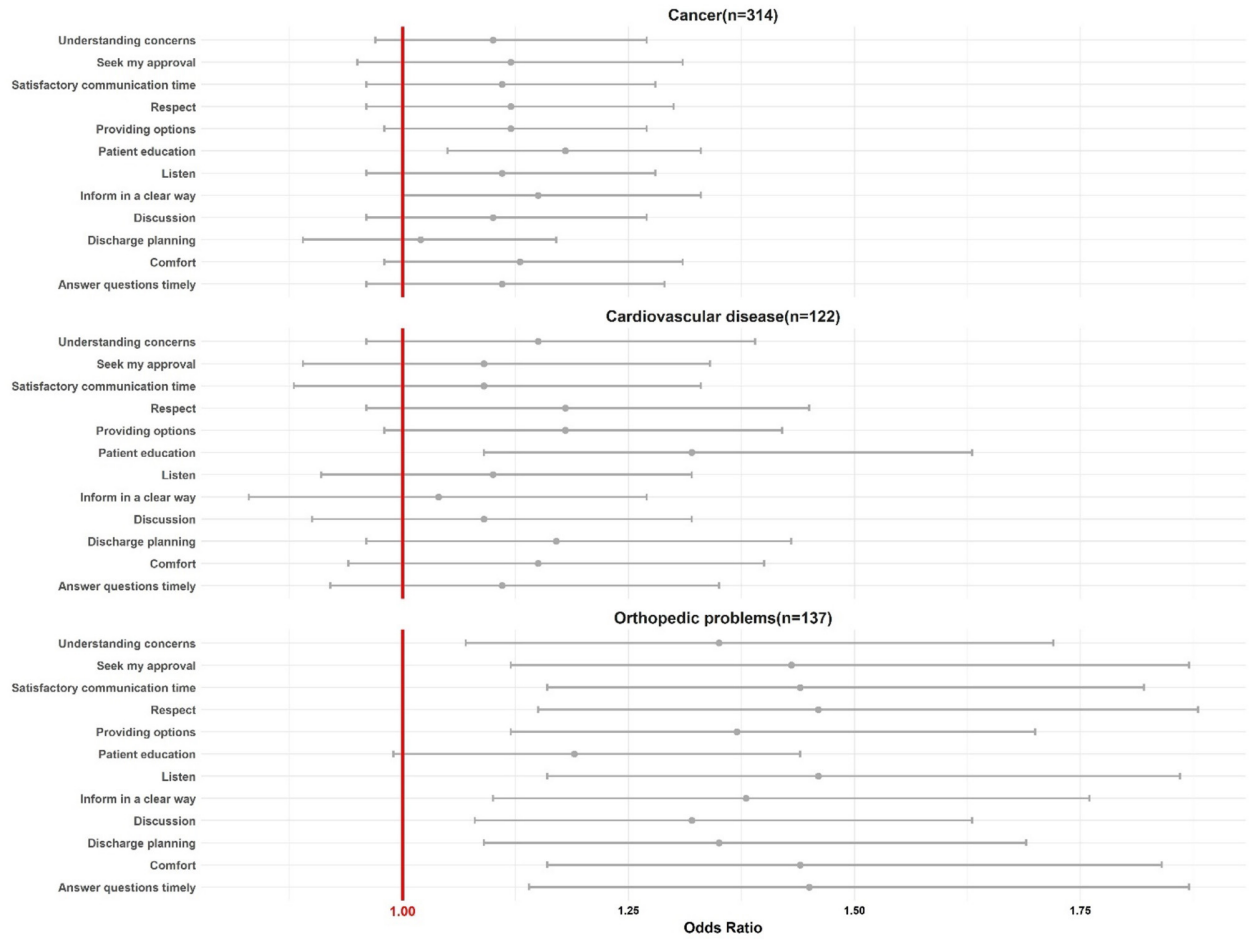
The odds ratio of developing depression predicted by satisfaction with PE among patients with the three most reported diseases.

The odds of developing depression predicted by satisfaction with PE among patients with the three most reported diseases was further estimated (Figure 2). Except for the item regarding patient education, patients with orthopedic problems were more likely to report having no depression when they showed high satisfaction with the other PE items. On the contrary, for patients with cancer or cardiovascular disease, only those who were satisfied with receiving sufficient patient education reported no significant depression (OR_cancer_ = 1.18, 95% CI_cancer_ = 1.05–1.33; OR_cardiovascular_ = 1.32, 95% CI_cardiovascular_ = 1.09–1.63).

## Discussion

This was the first study to use the PSM approach to minimize the confounding effects and selection bias of patients' chronic conditions to confirm that a high level of patient satisfaction with PE is associated with a low level of self-reported depression among hospitalized patients in China. Univariate analysis indicated that there was a significant relationship between all PE approaches and self-reported depression. Multivariate analysis further confirmed that patients satisfied with inpatient education programs that equipped them with sufficient knowledge and skills to manage health problems, and involvement in discharge planning, could significantly decrease their probability of developing depression. However, the effects of these two PE interventions to reduce depression were different, and patient education was more effective for urban patients, whereas rural patients benefited more from involvement in discharge planning. Although the concept of PE is complex, according to Carman et al.'s framework, PE is a process across different levels of the healthcare system (37); however, in this study, we only assessed the impact of PE on patients' depressive status from the level of direct care—effects of organizational governance, and policy making were not evaluated. Thus, although encouraging PE may indeed improve patients' mental health outcomes, if organizations and systems cannot support patients to make choices that align with their concerns, interests, and needs, the beneficial effect of PE on risk factors and health outcomes might be reduced.

Multivariate analysis demonstrated that, when all PE approaches were adjusted in this study, a satisfactory patient education program that provided the knowledge and skills to manage health problems could significantly reduce the odds of patients reporting depression due to hospitalization by 20%. This supported the findings of some previous studies. For example, Polat et al. found that providing colorectal cancer patients with knowledge of appropriate patient management, reduced anxiety and depression levels during the course of treatment (38). Kugbey et al. indicated that improving breast cancer survivors' health literacy and access to health information could reduce their levels of depression and anxiety (39). One of the primary goals of PE is to integrate patients as partners in the care team, to share decision-making, and improve health outcomes. To be successful, patients need to clearly express their preferences, values, and needs to medical providers, which heavily relies on patient education (1). When patients are equipped with sufficient knowledge about their disease and treatment options, they can better identify how they do or do not want to receive their healthcare. It can decrease the probability of generating un- or over-expected outcomes, reducing decisional regret and, in turn, avoiding depression or anxiety (40). However, challenges remain in providing patients with quality and accessible information to improve health and quality of life.

Involvement in discharge planning was an additional PE predictor that significantly estimated hospitalized patients' depressive status. Although a clear value to consider shared decision making within goal-setting for prognosis has been identified (41), efforts to investigate the relationship between PE in discharge planning and self-reported depression are limited. A systematic review indicated that the majority of included studies focused on investigating patients' motivation to engage in rehabilitation instead of evaluating the engagement *per se* (42). Empirical evidence in this study supported the fact that, despite relieving patients' depression by improving PE in discharge planning, this only occurred when patients' informed preferences in discharge planning had been fully understood and acknowledged, which was a challenge. The test for heterogeneity further demonstrated that involving patients in discharge planning could not decrease the probability of reporting a depressive status in patients with cancer and cardiovascular disease, but was effective for patients with orthopedic problems (non-chronic disease). Although previous studies indicated that detailed discharge planning could reduce anxiety and depression in cancer patients who underwent or completed treatment (43–45), our study confirmed the significant role of PE in this process. However, the heterogeneous effects of chronic and non-chronic diseases on this relationship suggest that further studies are needed.

Another interesting finding was that, in this study, urban patients who were satisfied with patient education were more likely to report a significantly low possibility of developing depression; however, the positive effect of involving patients in discharge planning was more significant in reducing depression in rural patients. The difference in satisfaction with PE interventions between urban and rural patients has not been studied in China or worldwide. Poor health literacy, widely associated with poor health, low quality of life, and impaired well-being (39, 46) could explain this difference, given that, in this study, 26.2% of urban patients had a university degree, compared with only 4.6% of rural patients. However, rural patients, limited by insurance reimbursement rates, geographical barriers, and social stigma (47, 48), reported a low possibility of developing depression when they were satisfied with their involvement in designing their discharge plan, which could minimize the likelihood of hospital readmission.

Several limitations of this study should be addressed. First, in this study, although PSM was used to control for covariates of PE approaches and patients' demographics, some unobserved variables, such as clinical indicators (not collected), were not included as covariates for the adjustment for balancing baseline characteristics between matched groups to reduce selection bias. Second, applying the PSM approach in this study resulted in more than 700 patients being excluded from the analysis, that may have led to information loss and affected the generalizability of the findings. Third, we did not collect information about the reasons for dropout, which may affect the reliability of our findings. Forth, we did not collect the information about the patients' history of mental health, thus, it is uncertain that the self-reported depression was developed during the hospitalization or not. Last, all PE items were elicited from the patient-centered care questionnaire. Despite several PE characteristics have been included in this survey, some other important characteristics may have been excluded, potentially affecting the validity of the findings. Further studies should be conducted to investigate the relationship between PE and mental health outcomes at different levels of the healthcare system, using PE-specific instruments.

## Conclusions

Clinical depression is a medical condition that can have a profound and long-lasting impact on patients' daily activities. In this study, we used the PSM approach to minimize the confounding effects of chronic conditions and confirmed that there is a positive association between improved patients' satisfaction with PE and decreased probability of developing a depressive status during hospitalization in China. However, heterogeneous PE preferences between urban and rural patients existed, that influenced the effectiveness of improving mental health. Based on our findings, areas of future research might include: (1) longitudinal studies of PE assessment using PE-specific measures for different patient groups to investigate the relationship between PE and patients' depression and other psychological distress; (2) studies measuring medical providers' attitudes and behaviors in implementing PE to improve patients' mental health; and (3) studies comparing the effectiveness of PE interventions on different levels of the healthcare system.

## Data Availability Statement

The raw data supporting the conclusions of this article will be made available by the authors, without undue reservation.

## Ethics Statement

The studies involving human participants were reviewed and approved by Ethics Committee of the Second Affiliated Hospital of Guangzhou Medical University. The patients/participants provided their written informed consent to participate in this study.

## Author Contributions

RX: study concept and design, data analysis and interpretation, software, writing-original draft, and writing-review and editing. L-mZ: study concept and design, data analysis and interpretation, and writing–review and editing. EW: provision of study materials or patients, supervision, and writing–review and editing. JC: study concept and design and writing–review and editing. DW: study concept and design, provision of study materials or patients, collection and assembly of data, supervision, and writing–review and editing. All authors contributed to the article and approved the submitted version.

## Funding

This study was funded by a grant from Guangdong Basic and Applied Basic Research Foundation (2021A1515011973), the Public Health Policy Research and Evaluation Key Laboratory Project of the Philosophy and Social Sciences of Guangdong College (2015WSYS0010), and the Public Health Service System Construction Research Foundation of Guangzhou, China (2021–2023).

## Conflict of Interest

The authors declare that the research was conducted in the absence of any commercial or financial relationships that could be construed as a potential conflict of interest.

## Publisher's Note

All claims expressed in this article are solely those of the authors and do not necessarily represent those of their affiliated organizations, or those of the publisher, the editors and the reviewers. Any product that may be evaluated in this article, or claim that may be made by its manufacturer, is not guaranteed or endorsed by the publisher.
