# Supplementary material for: Satisfaction With Patient Engagement and Self-Reported Depression Among Hospitalized Patients: A Propensity-Score Matching Analysis
Source: Front Psychiatry. 2022 Mar 9;13:751412. doi: 10.3389/fpsyt.2022.751412 (PMC8959894; doi:10.3389/fpsyt.2022.751412)
Supplement: Supplementary file 1 [file Data_Sheet_1.docx]

Table S1 the response rate in each hospital

| **Hospital/city** | **No. of patients invited** | **Questionnaire completed** | **Response rate** |
| --- | --- | --- | --- |
| Guangzhou | 1060 | 898 | 84.7 |
| Shenzhen | 628 | 431 | 68.6 |
| Shaoguan | 284 | 263 | 92.6 |
| Zhanjiang | 270 | 215 | 79.6 |
| Meizhou | 540 | 480 | 88.9 |
| Overall | 2782 | 2287 | 82.2 |

Table S2 Univariate and multivariate logistic regression analyses for predictors of depressive status for the all sample

|  | **Univariate analysis** | **Multivariate analysis** |
| --- | --- | --- |
|  | **Overall** | **Overall** |
|  | OR (95% C.I.) | OR (95% C.I.) |
| Informing all possible options | 1.157(1.096,1.222) ^***^ | 1.074(0.964,1.196) |
| Listening to my story | 1.151(1.086,1.221) ^***^ | 0.949(0.82,1.097) |
| Respecting my preferences | 1.17(1.1,1.244) ^***^ | 1.041(0.894,1.211) |
| Discussing medical plans with me | 1.166(1.1,1.235) ^***^ | 1.094(0.953,1.255) |
| Understanding my thoughts and concerns | 1.145(1.083,1.212) ^***^ | 0.994(0.874,1.126) |
| Comforting me when I feel stressed | 1.171(1.104,1.244) ^***^ | 1.115(0.988,1.257) |
| Communication time is satisfactory | 1.147(1.08,1.218) ^***^ | 0.952(0.817,1.108) |
| Telling me the disease progress in a clear way | 1.142(1.073,1.214) ^***^ | 0.958(0.816,1.12) |
| Seeking my approval before treatment start | 1.152(1.077,1.23) ^***^ | 0.976(0.842,1.131) |
| Answering my questions timely | 1.151(1.082,1.222) ^***^ | 1.027(0.894,1.178) |
| Providing sufficient patient education | 1.153(1.096,1.213) ^***^ | 1.125(1.032,1.225) ^**^ |
| Involving me in discharge planning | 1.117(1.054,1.183) ^***^ | 0.898(0.802,1.001) |
| Chronic condition (yes) | - | 0.639(0.516,0.788) ^***^ |
